# Supplementary material for: Improving residents’ satisfaction with administrative boundary changes: A comparative analysis based on the township-town merger policy
Source: PLoS One. 2026 Apr 15;21(4):e0346975. doi: 10.1371/journal.pone.0346975 (PMC13082704; doi:10.1371/journal.pone.0346975)
Supplement: S1 Table — (DOCX) [file pone.0346975.s002.docx]

**Table 1 Construction of policy effect evaluation indicators**

| **Primary Indicator** | **Secondary Indicator** |
| --- | --- |
| Population Development Effect | Population Size |
|  | Population Quality |
|  | Elderly Population (Negative Indicator) |
| Infrastructure Effect | Road Traffic Conditions |
|  | Water Resource Utilization and Management |
|  | Communication and Broadband Services |
| Environmental Improvement Effect | Sewage Treatment Facilities |
|  | Waste Disposal Facilities |
|  | Greening Status |
|  | Ecological Environment |
| Economic Growth Effect | Income Promotion |
|  | Employment Incentive |
| Social Security Effect | Medical and Health Conditions |
|  | Sports Fitness Equipment & Libraries |
|  | Public Security Situation |
|  | Life Convenience Level |
| Cultural Development Effect | Rational Utilization of Cultural Relics |
|  | Protection of Cultural Heritage Sites |
|  | Tourism Attractiveness |
|  |  |
