# Supplementary material for: Improving residents’ satisfaction with administrative boundary changes: A comparative analysis based on the township-town merger policy
Source: PLoS One. 2026 Apr 15;21(4):e0346975. doi: 10.1371/journal.pone.0346975 (PMC13082704; doi:10.1371/journal.pone.0346975)
Supplement: S2 Table — (DOCX) [file pone.0346975.s003.docx]

**Table 2 Robustness test**

|  | **(1)ologit** | **(2)** |
| --- | --- | --- |
|  | pg2 | Policy Satisfaction |
| Population Development Effect | 0.4846^*^ | 1.4674 |
|  | (0.2517) | (1.0641) |
| Infrastructure Effect | 0.1704 | 0.8355 |
|  | (1.8691) | (1.1361) |
| Environmental Improvement Effect | 1.0237 | 1.8232 |
|  | (3.6192) | (1.3627) |
| Income Growth Effect | -0.8324 | -1.0017 |
|  | (1.6544) | (0.7504) |
| Employment Incentive Effect | 0.3712^**^ | 1.3365^*^ |
|  | (0.1551) | (0.7497) |
| Social Security Effect | 2.6104^***^ | 3.1810^**^ |
|  | (0.1791) | (1.2979) |
| Cultural Development Effect | 3.4789^**^ | 2.5836^***^ |
|  | (1.5107) | (0.8838) |
| Gender | 0.3481 | 0.2399 |
|  | (0.4372) | (0.3566) |
| Age_18 | 0.8271 | 0.8045 |
|  | (1.1236) | (0.7810) |
| Age_31 | 0.9488 | 0.8339 |
|  | (0.5823) | (0.6456) |
| Age_46 | 0.7244^***^ | 1.0506^*^ |
|  | (0.2281) | (0.5549) |
| Occupation_Agriculture | -0.3560 | -0.5028 |
|  | (1.2713) | (0.6964) |
| Occupation_Firm | 0.0865 | -0.0467 |
|  | (0.1555) | (0.6448) |
| Occupation_Government | -0.3748 | -0.1617 |
|  | (0.4199) | (0.9093) |
| Occupation_Teaching | -1.2665 | -2.2256^**^ |
|  | (0.8910) | (0.9584) |
| Occupation_Free | -0.2674 | -0.3028 |
|  | (0.4657) | (0.6291) |
| ln_time | 0.0111 | 0.0751 |
|  | (0.0277) | (0.2442) |
|  |  |  |
| Month fixed effects | No | Yes |
| N | 243 | 243 |
| pseudo R^2^ | 0.179 | 0.350 |
