# Supplementary material for: Improving residents’ satisfaction with administrative boundary changes: A comparative analysis based on the township-town merger policy
Source: PLoS One. 2026 Apr 15;21(4):e0346975. doi: 10.1371/journal.pone.0346975 (PMC13082704; doi:10.1371/journal.pone.0346975)
Supplement: S3 Table — (DOCX) [file pone.0346975.s004.docx]

**Table 3 Principal components eigenvalues >1**

| **Component** | **Eigenvalue** | **Proportion** | **Cumulative** |
| --- | --- | --- | --- |
| Comp1 | 4.54301 | 0.2672 | 0.2672 |
| Comp2 | 1.81614 | 0.1068 | 0.3741 |
| Comp3 | 1.46592 | 0.0862 | 0.4603 |
| Comp4 | 1.25106 | 0.0736 | 0.5339 |
| Comp5 | 1.21096 | 0.0712 | 0.6051 |
| Comp6 | 1.09892 | 0.0646 | 0.6698 |
| Comp7 | 1.04422 | 0.0614 | 0.7312 |
