# Supplementary material for: Improving residents’ satisfaction with administrative boundary changes: A comparative analysis based on the township-town merger policy
Source: PLoS One. 2026 Apr 15;21(4):e0346975. doi: 10.1371/journal.pone.0346975 (PMC13082704; doi:10.1371/journal.pone.0346975)
Supplement: S4 Table — (DOCX) [file pone.0346975.s005.docx]

**Table 4 Fairlie decomposition intergroup differences (probit)**

|  | **Contribution Value** | **Standard Error** | **Contribution Percentage** |
| --- | --- | --- | --- |
| Population Development Effect | 0.0260 | 0.0202 | 0.1478 |
| Infrastructure Effect | 0.0017 | 0.0208 | 0.0097 |
| Environmental Improvement Effect | 0.0131 | 0.0486 | 0.0745 |
| Income Growth Effect | -0.0119 | 0.0146 | -0.0677 |
| Employment Incentive Effect | 0.0399*** | 0.0147 | 0.2269 |
| Social Security Effect | 0.0715*** | 0.0095 | 0.4065 |
| Cultural Development Effect | 0.0209*** | 0.0072 | 0.1188 |
