# Supplementary material for: Improving residents’ satisfaction with administrative boundary changes: A comparative analysis based on the township-town merger policy
Source: PLoS One. 2026 Apr 15;21(4):e0346975. doi: 10.1371/journal.pone.0346975 (PMC13082704; doi:10.1371/journal.pone.0346975)
Supplement: S5 Table — (DOCX) [file pone.0346975.s006.docx]

**Table 5 Fairlie decomposition: Taowu vs. Lukou (probit)**

|  | **Coefficient** | **Contribution Percentage** |
| --- | --- | --- |
| Policy Satisfaction - Lukou | 0.5609 |  |
| Policy Satisfaction - Taowu | 0.4347 |  |
| Total difference | 0.1262 |  |
| Population Development Effect | 0.1055*** | 0.8359 |
| Infrastructure Effect | -0.0142 | -0.1125 |
| Environmental Improvement Effect | 0.0766 | 0.6069 |
| Income Growth Effect | -0.0464 | -0.3677 |
| Employment Incentive Effect | 0.0418*** | 0.3312 |
| Social Security Effect | 0.0609*** | 0.4826 |
| Cultural Development Effect | 0.0076 | 0.0602 |
