# Supplementary material for: Improving residents’ satisfaction with administrative boundary changes: A comparative analysis based on the township-town merger policy
Source: PLoS One. 2026 Apr 15;21(4):e0346975. doi: 10.1371/journal.pone.0346975 (PMC13082704; doi:10.1371/journal.pone.0346975)
Supplement: S6 Table — (DOCX) [file pone.0346975.s007.docx]

**Table 6 Policy satisfaction: Taowu vs. Hengxi (probit)**

|  | **Coefficient** | **Contribution Percentage** |
| --- | --- | --- |
| Policy Satisfaction - Hengxi | 0.3652 |  |
| Policy Satisfaction - Taowu | 0.4347 |  |
| Total difference | -0.0695 |  |
| Population Development Effect | 0.0009 | -0.0129 |
| Infrastructure Effect | -0.0049 | 0.0705 |
| Environmental Improvement Effect | -0.0260** | 0.3741 |
| Income Growth Effect | 0.0011 | -0.0158 |
| Employment Incentive Effect | 0.0238*** | -0.3425 |
| Social Security Effect | 0.0202* | -0.2907 |
| Cultural Development Effect | -0.0120 | 0.1727 |
