# Supplementary material for: Improving residents’ satisfaction with administrative boundary changes: A comparative analysis based on the township-town merger policy
Source: PLoS One. 2026 Apr 15;21(4):e0346975. doi: 10.1371/journal.pone.0346975 (PMC13082704; doi:10.1371/journal.pone.0346975)
Supplement: S7 File — (DOCX) [file pone.0346975.s008.docx]

**Informed Consent Statement**

Dear Residents,

To gain an in-depth understanding of the impact of the township merger policy on local development, we are conducting this survey. This questionnaire aims to collect your views and feelings regarding changes in the following areas since the merger of townships in 2006: Population status, Infrastructure, Economic development, Resident employment, Public services and social programs, Preservation of traditional culture.

The insights gathered will help us better analyze the effects of the policy and provide references for the sustainable development of our locality.

The questionnaire is anonymous and does not collect personally identifiable information such as your name, address, or ID number. Please answer the questions truthfully. Participation is entirely voluntary, and you have the right to refuse to participate or withdraw at any time during the survey.

We assure you that all information provided will be kept strictly confidential and used solely for the purpose of this research.

Thank you for your support and cooperation!

If you have any questions, please contact:

[Wang Qiong / Nanjing Xiaozhuang University]

Phone: [13851820101]

□ I have read and agree to participate.
