# Supplementary material for: Improving residents’ satisfaction with administrative boundary changes: A comparative analysis based on the township-town merger policy
Source: PLoS One. 2026 Apr 15;21(4):e0346975. doi: 10.1371/journal.pone.0346975 (PMC13082704; doi:10.1371/journal.pone.0346975)
Supplement: S8 File — (PDF) [file pone.0346975.s009.pdf]

Request for Waiver of Ethical Review

This study intends to invite residents from the three towns of Lukou, Taowu, and Hengxi in the Jiangning District of Nanjing to complete a questionnaire. It aims to investigate their subjective perceptions, satisfaction levels, and non-sensitive suggestions regarding the Township-town merger policy, particularly in terms of public services, life convenience, and economic development.

1. This research constitutes an anonymous questionnaire survey within the field of social sciences. The questionnaire does not record identifiable information such as name, ID number, or residential address. It does not involve biomedical experiments, the collection of sensitive personal information, the imposition of psychological stress, or any form of interventional procedures.

2. Prior to conducting the research, informed consent forms have been attached to both the online and offline questionnaires. Participants must explicitly check the option stating "I have read and understood the above information and voluntarily agree to participate in this survey" before proceeding to the questionnaire section. This process ensures that participation is entirely voluntary and informed.

3. This study entails minimal anticipated risk. It does not involve minors, will not subject participants to any form of deception or physical/psychological harm, and implements thorough anonymization and confidentiality measures, resulting in an extremely low risk of privacy leakage.

Based on the above, the study aligns with the general conditions for exemption from ethical review as commonly adopted in both national and international practices and can therefore be exempted from ethical review.

Applicant: [illegible]

College of Foreign Languages, Nanjing Xiaozhuang University）

Date: 2024/09/08

南京晓庄学院 外国语学院
