# Supplementary material for: Improving residents’ satisfaction with administrative boundary changes: A comparative analysis based on the township-town merger policy
Source: PLoS One. 2026 Apr 15;21(4):e0346975. doi: 10.1371/journal.pone.0346975 (PMC13082704; doi:10.1371/journal.pone.0346975)
Supplement: Attachment — Submitted filename: Response to Reviewers.docx [file pone.0346975.s013.docx]

***Response to editor and reviewers***

**Dear Editor and reviewers:**

Thanks for your helpful recommendations. This is a revised manuscript with the title "*Improving residents' satisfaction with administrative boundary changes: A comparative analysis based on the township-town merger policy* "(PONE-D-25-65675). We have revised the manuscript thoroughly according to your comments and carefully proofread the manuscript to minimize typographical and grammatical errors.

Moreover, we attached the revised manuscript in the format of WORD for your approval. A document answering every question from you and the reviewers was also summarized and enclosed.

A revised manuscript with the correction sections red-marked was attached as the supplemental material for easy checking purposes.

Should you have any questions, please contact us without hesitation. Thank you again for your constructive suggestions!

***Editor :***

1. **Please ensure that your manuscript meets PLOS ONE's style requirements, including those for file naming.**

**Answer:** We are sure that our manuscript meets PLOS ONE's style requirements, including those for file naming.

2. **We note that the data set contains text or data that is not in English. Please note that PLOS is an English-language publisher, so we require data sets to be provided in English as well. Please Please upload an English-language version of your data set.**

**Answer:** We removed the Chinese versions of the informed consent statement and the request for an ethical waiver statement from the supporting information, retaining only their English versions in the data set.

3. **We note that Figure 1 in your submission contain [map/satellite] images which may be copyrighted. All PLOS content is published under the Creative Commons Attribution License (CC BY 4.0), which means that the manuscript, images, and Supporting Information files will be freely available online, and any third party is permitted to access, download, copy, distribute, and use these materials in any way, even commercially, with proper attribution. For these reasons, we cannot publish previously copyrighted maps or satellite images created using proprietary data, such as Google software (Google Maps, Street View, and Earth). For more information, see our copyright guidelines: http://journals.plos.org/plosone/s/licenses-and-copyright.**

**Answer:** We uploaded a new figure to replace the old one. The new figure, which is not copyrighted, is free for everyone to download.

**Action：**We download the new figure at Natural Earth (public domain): http://www.naturalearthdata.com/

4. **Please include captions for your Supporting Information files at the end of your manuscript, and update any in-text citations to match accordingly. Please see our Supporting Information guidelines for more information: http://journals.plos.org/plosone/s/supporting-information.**

**Answer:** We have added captions for our Supporting Information files at the end of our manuscript and updated any in-text citations to match accordingly. As seen in our manuscript, each file in the Supporting Information has been assigned numbers and types to them in the list.

***Reviewer #1:***

1. **Several technical aspects require clarification, particularly regarding the justification for setting satisfaction coding thresholds (a score of 1-3 for dissatisfied and 4-5 for satisfied), as well as potential sampling bias due to the combination of online and offline surveys and the relatively long data collection period.**

**Answer:** Thank you for your suggestion. The comments raised two issues: the appropriateness of the variable settings and potential sample bias. We will address them accordingly.

**(1) Rationale for Classification:** 1) **First**, this classification is founded on data characteristics (Question item design: in the questionnaire, 1-5 represents different levels of opinion, where 1-3 indicates opposition and 4-5 indicates support. Assigning 3 points to "dissatisfied" prevents overestimating satisfaction).

**Second**, the coding threshold for satisfaction was set based on academic conventions and similar studies in policy satisfaction research.

Our classification is consistent with the common practice of dichotomizing Likert-scale data in policy evaluation studies (Gu et al., 2021; Liu, 2014; Wang et al., 2019). For example, Gu et al. (2021) used a 5-point scale to measure farmers' satisfaction with the Link Policy and categorized 1–2 as "dissatisfied" and 4–5 as "satisfied"; Liu (2014) adopted the same threshold setting in evaluating Taiwan's National Health Insurance policy, arguing that the midpoint (3 points) of the 5-point scale represents a neutral attitude and can be reasonably grouped with negative evaluations when focusing on "satisfaction vs. Dissatisfaction" dichotomy. Additionally, Pelletier et al. (1996) used a 7-point scale in environmental satisfaction research and similarly classified the first half of the scale as negative evaluations and the second half as positive, supporting the rationality of our threshold setting.

**Third,** we use an alternative dependent variable as a robustness check. Specifically, we measure policy satisfaction on a 1-5 scale, where higher scores indicate greater satisfaction. In terms of model specification, because the dependent variable is an ordered discrete value, we re-estimate the regression using an ordered logit model. The results are presented in Table 1. Column (1) shows the baseline regression results, and Column (2) reports the results of the ordered logit model. A comparison between the two reveals that the employment incentive effect, social security effect, and cultural development effect still have a positive impact on residents' policy satisfaction at the 5% significance level, confirming the appropriateness of the variable specification.

**Table 1**

|  | **(1)logit** | **(2) ologit** |
| --- | --- | --- |
|  | Policy Satisfafction | Policy Satisfaction_alter |
|  |  |  |
| Population Development Effect | 1.0650 | 0.4846^*^ |
|  | (0.8544) | (0.2517) |
| Infrastructure Effect | 0.3068 | 0.1704 |
|  | (2.3137) | (1.8691) |
| Environmental Improvement Effect | 0.6916 | 1.0237 |
|  | (3.2677) | (3.6192) |
| Income Growth Effect | -0.9425 | -0.8324 |
|  | (1.2393) | (1.6544) |
| Employment Incentive Effect | 1.3958^**^ | 0.3712^**^ |
|  | (0.5538) | (0.1551) |
| Social Security Effect | 2.8941^***^ | 2.6104^***^ |
|  | (0.0870) | (0.1791) |
| Cultural Development Effect | 2.8488^**^ | 3.4789^**^ |
|  | (1.1858) | (1.5107) |
| Gender | 0.1714 | 0.3481 |
|  | (0.4103) | (0.4372) |
| Age_18 | 0.6904 | 0.8271 |
|  | (0.8645) | (1.1236) |
| Age_31 | 0.5499^*^ | 0.9488 |
|  | (0.3179) | (0.5823) |
| Age_46 | 0.6235^**^ | 0.7244^***^ |
|  | (0.2798) | (0.2281) |
| Occupation_Agriculture | -0.3812 | -0.3560 |
|  | (0.5998) | (1.2713) |
| Occupation_Firm | 0.1475^***^ | 0.0865 |
|  | (0.0154) | (0.1555) |
| Occupation_Government | 0.5716 | -0.3748 |
|  | (0.3813) | (0.4199) |
| Occupation_Teaching | -1.2339 | -1.2665 |
|  | (0.8246) | (0.8910) |
| Occupation_Free | -0.2226 | -0.2674 |
|  | (0.4515) | (0.4657) |
| ln_time | 0.1699^*^ | 0.0111 |
|  | (0.0883) | (0.0277) |
| _cons | -6.5936^***^ |  |
|  | (1.2857) |  |
| N | 243 | 243 |
| Pseudo R^2^ | 0.306 | 0.179 |

**Action:** we use an alternative dependent variable as a robustness check. Specifically, we measure policy satisfaction on a 1-5 scale, where higher scores indicate greater satisfaction. In terms of model specification, because the dependent variable is an ordered discrete value, we re-estimate the regression using an ordered logit model. The results are presented in Table A1. The result reveals that the employment incentive effect, social security effect, and cultural development effect still have a positive impact on residents' policy satisfaction at the 5% significance level, confirming the appropriateness of the variable specification.

**Action place:** "Results" section

(2) **Sample bias: the combination of online and offline surveys.** We used both online and offline surveys to meet the needs of different surveyors. While younger demographics show higher demand for online platforms, some elderly participants primarily require offline surveys due to their limited access to digital devices. Although 50 individuals are taking the offline surveys, which accounts for a small proportion of the whole participants, we acknowledge that this could introduce bias into our data collection process.

To address this issue, we **first** included age groups as control variables to partially mitigate the impact of selection bias among older respondents.

**Figure 1**

**Second**, given the difference between younger and older individuals in their ability to use digital devices and their cognitive abilities, we used both online and offline survey methods. Accordingly, we attempted to control for this factor in our model to eliminate its potential interference with the baseline conclusions. Generally, as age increases, an individual's cognitive ability and adaptability to new things gradually decline. In the context of this study, the time spent answering questions using electronic devices also increases with age (see Figure 1). Therefore, we use the logarithm of the time spent completing the questionnaire as a proxy variable for individuals' digital device proficiency and cognitive ability. After incorporating this variable into the baseline model, the regression results are shown in Table 2. The coefficients and significance levels of the core independent variables did not change substantially, confirming the robustness of our conclusions.

In the revised manuscript, after incorporating this cognitive variable as a control variable in the baseline model, we re-estimated all regressions, and the results remained robust.

**Table 2**

|  | **(1)** | **(2)** |
| --- | --- | --- |
|  | Policy Satisfafction | Policy Satisfafction |
| Population Development Effect | 1.4119^**^ | 1.0650 |
|  | (0.7184) | (0.8544) |
| Infrastructure Effect | 0.4057 | 0.3068 |
|  | (2.0788) | (2.3137) |
| Environmental Improvement Effect | 0.2285 | 0.6916 |
|  | (3.5689) | (3.2677) |
| Income Growth Effect | -0.6951 | -0.9425 |
|  | (1.2467) | (1.2393) |
| Employment Incentive Effect | 1.4578^***^ | 1.3958^**^ |
|  | (0.5100) | (0.5538) |
| Social Security Effect | 2.2970^***^ | 2.8941^***^ |
|  | (0.3480) | (0.0870) |
| Cultural Development Effect | 3.1524^**^ | 2.8488^**^ |
|  | (1.2752) | (1.1858) |
| Gender |  | 0.1714 |
|  |  | (0.4103) |
| Age_18 |  | 0.6904 |
|  |  | (0.8645) |
| Age_31 |  | 0.5499^*^ |
|  |  | (0.3179) |
| Age_46 |  | 0.6235^**^ |
|  |  | (0.2798) |
| Occupation_Agriculture |  | -0.3812 |
|  |  | (0.5998) |
| Occupation_Firm |  | 0.1475^***^ |
|  |  | (0.0154) |
| Occupation_Government |  | 0.5716 |
|  |  | (0.3813) |
| Occupation_Teaching |  | -1.2339 |
|  |  | (0.8246) |
| Occupation_Free |  | -0.2226 |
|  |  | (0.4515) |
| ln_time |  | 0.1699^*^ |
|  |  | (0.0883) |
| _cons | -5.0998^***^ | -6.5936^***^ |
|  | (0.0883) | (1.2857) |
| N | 243 | 243 |
| Pseudo R^2^ | 0.285 | 0.306 |

**Action:** Individual characteristics considered in the study include gender, age, and occupation and cognitive ability. Gender is coded as a binary variable, with males assigned a value of 1 and females a value of 0. Age is categorized into four groups: 18–30 years, 31–45 years, 46–60 years, and over 60 years, with the oldest group serving as the reference category; three dummy variables represent the remaining age groups. Occupation is classified into six categories: enterprise employee, farmer, government staff, teacher, self-employed, and retiree. Retirees are used as the reference group, and five dummy variables are constructed to capture the other occupational categories. Considering that cognitive abilities and the capacity to adapt to new things gradually decline with age among the elderly, we use the logarithm of the time (in seconds) spent on completing the questionnaire as a proxy variable for measuring the cognitive abilities of the elderly. The longer the time taken to complete the questionnaire, the lower their cognitive abilities.

Additionally, we also made corresponding adjustments in all regression results. For example, in Table 3, cognitive ability was included in the model as a control variable.

**Table 3**

|  | **(1)** | **(2)** | **(3)** | **(4)** | **(5)** |
| --- | --- | --- | --- | --- | --- |
|  | Policy Satisfafction | Policy Satisfafction | Policy Satisfafction | Policy Satisfafction | Margin effects |
| Population Development Effect | 1.4119^**^ | 1.0650 | 1.0650 | 1.0650 | 0.259 |
|  | (0.7184) | (0.8544) | (1.0078) | (1.2489) | (0.211) |
| Infrastructure Effect | 0.4057 | 0.3068 | 0.3068 | 0.3068 | 0.075 |
|  | (2.0788) | (2.3137) | (1.0795) | (1.3809) | (0.562) |
| Environmental Improvement Effect | 0.2285 | 0.6916 | 0.6916 | 0.6916 | 0.168 |
|  | (3.5689) | (3.2677) | (1.2713) | (1.4542) | (0.797) |
| Income Growth Effect | -0.6951 | -0.9425 | -0.9425 | -0.9425 | -0.229 |
|  | (1.2467) | (1.2393) | (0.7629) | (0.9470) | (0.304) |
| Employment Incentive Effect | 1.4578^***^ | 1.3958^**^ | 1.3958^*^ | 1.3958^***^ | 0.340^***^ |
|  | (0.5100) | (0.5538) | (0.7662) | (0.4712) | (0.131) |
| Social Security Effect | 2.2970^***^ | 2.8941^***^ | 2.8941^**^ | 2.8941^***^ | 0.705^***^ |
|  | (0.3480) | (0.0870) | (1.2555) | (1.0764) | (0.013) |
| Cultural Development Effect | 3.1524^**^ | 2.8488^**^ | 2.8488^***^ | 2.8488^***^ | 0.694^**^ |
|  | (1.2752) | (1.1858) | (0.8574) | (0.6170) | (0.281) |
| Gender |  | 0.1714 | 0.1714 | 0.1714 | 0.042 |
|  |  | (0.4103) | (0.3454) | (0.3117) | (0.099) |
| Age_18 |  | 0.6904 | 0.6904 | 0.6904 | 0.168 |
|  |  | (0.8645) | (0.7404) | (0.8243) | (0.209) |
| Age_31 |  | 0.5499^*^ | 0.5499 | 0.5499 | 0.134^*^ |
|  |  | (0.3179) | (0.6105) | (0.6745) | (0.076) |
| Age_46 |  | 0.6235^**^ | 0.6235 | 0.6235 | 0.152^**^ |
|  |  | (0.2798) | (0.5281) | (0.5376) | (0.070) |
| Occupation_Agriculture |  | -0.3812 | -0.3812 | -0.3812 | -0.093 |
|  |  | (0.5998) | (0.6477) | (0.9345) | (0.147) |
| Occupation_Firm |  | 0.1475^***^ | 0.1475 | 0.1475 | 0.036^***^ |
|  |  | (0.0154) | (0.6106) | (0.7250) | (0.003) |
| Occupation_Government |  | 0.5716 | 0.5716 | 0.5716 | 0.139 |
|  |  | (0.3813) | (0.8432) | (0.8372) | (0.091) |
| Occupation_Teaching |  | -1.2339 | -1.2339 | -1.2339 | -0.300 |
|  |  | (0.8246) | (0.9029) | (1.3728) | (0.197) |
| Occupation_Free |  | -0.2226 | -0.2226 | -0.2226 | -0.054 |
|  |  | (0.4515) | (0.5881) | (0.7847) | (0.109) |
| ln_time |  | 0.1699^*^ | 0.1699 | 0.1699 | 0.041^**^ |
|  |  | (0.0883) | (0.2229) | (0.3068) | (0.021) |
| _cons | -5.0998^***^ | -6.5936^***^ | -6.5936^***^ | -6.5936^***^ |  |
|  | (0.0883) | (1.2857) | (1.6776) | (1.4926) |  |
| N | 243 | 243 | 243 | 243 |  |
| pseudo R^2^ | 0.285 | 0.306 | 0.306 | 0.306 |  |

Note:* p < 0.1, ** p < 0.05, *** p < 0.01. Column 1 and Column 2 use cluster-level regional mislabeling, Column 3 uses standard error, and Column 4 uses a dual standard error for both region and time.

(3) **Relatively long data collection period:** We spent six months (from September 28th 2024 to March 16^th^ 2025) collecting data three times to compensate for the limited data resources from the elderly, who have restricted access to digital devices. During this period, there were no disruptions caused by external policies or shocks. To minimize the possible influence on the research results caused by our comparatively long data collection period, we controlled for month fixed effects in the model to mitigate time-specific shocks and potential biases across different survey waves. The regression results are presented in Table 4. The coefficients and significance levels of the core independent variables did not change substantially, confirming the robustness of our conclusions.

**Table 4**

|  | **(1)** |
| --- | --- |
|  | Policy Satisfaction |
| Population Development Effect | 1.4674 |
|  | (1.0641) |
| Infrastructure Effect | 0.8355 |
|  | (1.1361) |
| Environmental Improvement Effect | 1.8232 |
|  | (1.3627) |
| Income Growth Effect | -1.0017 |
|  | (0.7504) |
| Employment Incentive Effect | 1.3365^*^ |
|  | (0.7497) |
| Social Security Effect | 3.1810^**^ |
|  | (1.2979) |
| Cultural Development Effect | 2.5836^***^ |
|  | (0.8838) |
| Gender | 0.2399 |
|  | (0.3566) |
| Age_18 | 0.8045 |
|  | (0.7810) |
| Age_31 | 0.8339 |
|  | (0.6456) |
| Age_46 | 1.0506^*^ |
|  | (0.5549) |
| Occupation_Agriculture | -0.5028 |
|  | (0.6964) |
| Occupation_Firm | -0.0467 |
|  | (0.6448) |
| Occupation_Government | -0.1617 |
|  | (0.9093) |
| Occupation_Teaching | -2.2256^**^ |
|  | (0.9584) |
| Occupation_Free | -0.3028 |
|  | (0.6291) |
| ln_time | 0.0751 |
|  | (0.2442) |
|  |  |
| Month fixed effects | Yes |
| N | 243 |
| pseudo R^2^ | 0.350 |

**Action:**

We conducted robustness checks for the baseline regression. First, we use an alternative dependent variable as a robustness check. Specifically, we measure policy satisfaction on a 1-5 scale, where higher scores indicate greater satisfaction. In terms of model specification, because the dependent variable is an ordered discrete value, we re-estimate the regression using an or-dered logit model. The results are presented in Column (1) of Table A1. The result reveals that the employment incentive effect, social security effect, and cultural development effect still have a positive impact on residents' policy satisfaction at the 5% significance level, confirming the appropriateness of the variable specification.

Second, we included month fixed effects in the model. These fixed effects help rule out the influence of time-specific events on the policy evaluations of older adults, while also mitigating sample selection bias arising from different survey periods. The results are shown in Column (2) of Table A1, and the baseline conclusions remain robust.

**Action Place:** "Results" section

2. **First, regarding the conceptualization and measurement of policy satisfaction. Although the theoretical framework used draws on expectancy-performance theory and identity-based mechanisms, the operationalization of the dependent variable is based solely on a single global question regarding whether the policy "encourages local development."This approach is less in line with the multidimensional nature of policy satisfaction, which encompasses cognitive evaluations, affective responses, and perceptions of distributive justice.**

**Answer:** Thank you for your suggestions. In the existing literature, there is controversy regarding how to measure subjective indicators such as satisfaction or well-being. As you mentioned, satisfaction has multidimensional implications. However, compared to multidimensional measurement, single-indicator measurement offers the following advantages (Helliwell et al., 2026)

(1) Multidimensional composite indices typically encompass factors such as cognitive evaluations, affective responses, and perceptions of distributive justice. These are causes of satisfaction, rather than satisfaction itself. In contrast, a single-item measure directly captures satisfaction as an outcome.
(2) When "satisfaction" becomes a goal for policymakers, a clear overarching objective is often required to compare different policy options, and a single measurement metric is more suitable for such analysis. Moreover, policy satisfaction, at its essence, centers on citizens' perceptions of whether the core policy goal is achieved (Van Ryzin, 2004; Wang et al., 2019). For our study, "encouraging local development" directly corresponds to the primary objective of the policy, and a single-item measure can effectively capture the core evaluation without redundant dimensions.
(3) Single indicators are easy to collect and suitable for multi-regional comparisons.
(4) The ranking of composite indices may reflect the weights assigned by researchers to various factors rather than the genuine self-evaluation of individuals regarding their own lives. In contrast, single indicators are independent of subjective judgments by researchers, thereby avoiding bias.
(5) Existing empirical studies have widely adopted single-item measurements for policy satisfaction when the policy goal is clear and focused. For example, Wang et al. (2019) used a single 5-point Likert-scale to measure farmers' satisfaction with the Prohibited Grazing Policy, and Pelletier et al. (1996) validated that a single-item measure of government environmental policy satisfaction is strongly correlated with pro-environmental behaviors. Similarly, Rahmqvist & Bara (2010) used "overall satisfaction with medical encounters" as the single core indicator, focusing on the fundamental goal of "whether medical services meet healthcare needs."

(6) Moreover, we adopt the methodological logic of "single core indicator + multi-control variables". In this paper, we controlled individual characteristics such as gender, age, occupation, cognitive ability and so on, which, to some extent, inhibited the interference of confounding factors on the conclusion of this paper. This is consistent with the academic practice of previous influential studies (Fiorio & Florio, 2011; Rahmqvist & Bara 2010; Jilke & Van de Walle 2013).

**Action：**The dependent variable is a binary indicator measuring residents' satisfaction with the pol-icy, derived from the survey question: "Do you believe the township-town merger policy has significantly promoted local development?" Responses scored between 1 and 3 were coded as 0, representing lower satisfaction, while scores of 4 or 5 were coded as 1, representing higher satisfaction. In our study, the operationalization of the dependent variable is based solely on a single global question regarding whether the policy encourages local development. It is because that a single-item measure directly captures satisfaction as an outcome while avoiding the subjective bias introduced by weighting multidimensional indicators. Therefore, following Fiorio & Florio (2011)[41]?, we measure residents' policy satisfaction using a single-item measure.

Moreover, in this study, we controlled for individual characteristics to mitigate the inter-ference of confounding factors on the conclusions to some extent. Individual characteristics considered in the study include gender, age, and occupation and cognitive ability. Gender is coded as a binary variable, with males assigned a value of 1 and females a value of 0. Age is categorized into four groups: 18-30 years, 31-45 years, 46-60 years, and over 60 years, with the oldest group serving as the reference category; three dummy variables represent the remaining age groups. Occupation is classified into six categories: enterprise employee, farmer, government staff, teacher, self-employed, and retiree. Retirees are used as the reference group, and five dummy variables are constructed to capture the other occupational categories.With the aging of the elderly, their cognitive abilities and the ability to accept new things gradually decline. Therefore, this paper uses the logarithm of the time (seconds) spent completing the questionnaire as a proxy variable for cognitive ability in the elderly. The longer the time spent completing the questionnaire, the lower the corresponding cognitive ability.

**Action Place:** "Research Design" section

3. **There are potential issues of endogeneity and common method bias because the dependent and main independent variables are both measured through respondents' perceptions in a single survey instrument. The current manuscript does not acknowledge or discuss this limitation, nor does it implement methodological mitigation measures. Authors should explicitly address this potential bias and explain its implications for coefficient estimation and interpretation of results, or include relevant robustness checks where possible.**

**Answer :** We sincerely appreciate your valuable comments regarding the potential issues of endogeneity and common method bias, which may arise from measuring both dependent and independent variables through respondents' perceptions in a single survey instrument.

To mitigate these concerns, we implemented several ex-ante strategies during the survey design and data collection process. First, we collected data across multiple regions and at different time points to reduce the likelihood of consistent or patterned responses, thereby minimizing common method bias. Second, we ensured that the survey was conducted anonymously, encouraging respondents to provide truthful answers and reducing social desirability bias. Third, we randomized the order of questionnaire items to mitigate potential order effects and enhance measurement accuracy.

In addition to these procedural remedies, we conducted interviews with respondents to gain deeper insights into their actual perceptions of the policy. These qualitative findings helped us develop psychological mechanisms to explain cross-regional differences in policy satisfaction, thereby further strengthening the credibility of our conclusions.

We fully recognize that, given the constraints of our data (a single-wave questionnaire designed to capture citizens' perceptions of the Township-town Merger Policy), our ability to implement causal identification strategies is limited. Accordingly, we have made the following revisions in the revised manuscript: (1) We now emphasize linkages rather than causal relationships when interpreting the results. (2) We have added a discussion of this methodological limitation in the end of the manuscript.

To statistically assess common method bias, we conducted Harman's one-factor test (Andersson & Bateman, 1997; Aulakh & Gencturk, 2000). Specifically, as shown in the exploratory factor analysis results in Table 5, the first principal component accounts for 26.72% of the total variance. This value is substantially lower than the conventional threshold of 50% (Gong et al., 2022), suggesting that common method bias is not a serious concern in this study.

**Table 5**

| Component | Eigenvalue | Proportion | Cumulative |
| --- | --- | --- | --- |
| Comp1 | 4.54301 | 0.2672 | 0.2672 |
| Comp2 | 1.81614 | 0.1068 | 0.3741 |
| Comp3 | 1.46592 | 0.0862 | 0.4603 |
| Comp4 | 1.25106 | 0.0736 | 0.5339 |
| Comp5 | 1.21096 | 0.0712 | 0.6051 |
| Comp6 | 1.09892 | 0.0646 | 0.6698 |
| Comp7 | 1.04422 | 0.0614 | 0.7312 |

**Action:**

(1) To statistically assess common method bias, we conducted Harman's one-factor test (Andersson & Bateman, 1997; Aulakh & Gencturk, 2000). Specifically, as shown in the exploratory factor analysis results in Table A2, the first principal component accounts for 26.72% of the total variance. This value is substantially lower than the conventional threshold of 50% (Gong et al., 2022), suggesting that common method bias is not a serious concern in this study.

(2) Despite the insights obtained from this study, several methodological limitations need to be recognized. Endogeneity problems arising from the restricted design of questionnaire items and unobserved variables may impede strict causal inference in our study. Hence, when interpreting the model results, we placed more emphasis on the correlations among variables.

**Action Place:** (1) "Variables" section in the "research design" ; (2) "Limitation" section;

4. **Interpretations of psychological mechanisms such as identity recognition, expectancy formation, and social comparison have not been supported by direct measurement or modeling of these constructs. Therefore, claims regarding these mechanisms should be clearly distinguished between empirical findings and theoretical interpretations. Authors are advised to formulate these mechanisms as plausible explanations, not as empirically confirmed mechanisms.**

**Answer :** Thank you for your suggestions. **First,** in stead of directly measuring psychological mechanisms such as identity, expectation formation, and social comparison, we analyzed these mechanisms based on qualitative data from post-survey interviews. As described in Section "Questionnaire design and data collection" and "Analysis of the determinants of policy satisfaction", we conducted random offline interviews with respondents after the questionnaire survey. Drawing on these interviews, we explored the reasons underlying regional differences in residents' policy satisfaction.

**Second,** the existing literature supports the validity of deriving psychological mechanisms from interview data. As emphasized by Mettler & Soss (2004), qualitative evidence, such as citizen interviews, is a critical complement to quantitative analysis in public policy research, enabling the unpacking of the "how" and "why" behind observed patterns. Consistent with this, Rahmqvist & Bara (2010) and Fiorio & Florio (2011) both integrated interview insights to explain the psychological drivers of policy satisfaction (e.g., identity and social comparison), demonstrating that interviews can effectively capture subjective mechanisms that are not directly measurable via questionnaires.

**Moreover,** social identity theory (Tajfel & Turner, 1979) and social comparison theory (Wills, 1981), which are cited in our literature review, provide theoretical frameworks that align with our interview-derived interpretations, further validating their plausibility.

**Third,** in the original manuscript, both empirical findings and theoretical interpretations were placed in the "Results" section, which may have compromised the readability of the mechanism analysis. Therefore, we added a "Discussion" section to the revised manuscript to explicitly link the mechanisms to interview evidence and theoretical interpretation, while leaving the "Results" section solely for presenting statistical findings.

**Action : Psychological mechanisms**

In the comparison between Taowu and Lukou, there is a substantial gap in policy satisfaction (Table 6). This indicates that Taowu underperforms compared to Lukou in overall economic aspects, especially in population development, social security, and employment incentives. On one hand, these findings are consistent with Lai[12], who discovered that township mergers led to notable population agglomeration, which ultimately enhances residents' policy satisfaction. On the other hand, the interviews also reveal that in Lukou, not only is the population more concentrated, but the area has also attracted numerous migrants who are satisfied with the TMP. For instance, one interviewee stated that her son chose to settle and work in Lukou because of the city's well-organized job fairs and effective job-seeking WeChat public accounts, which means good job opportunities. Meanwhile, interviews with the residents of Taowu show that they are dissatisfied with the significant difference in the development process between the two places.

Q: "Are you satisfied with the development of Taowu after the township merger?"

A: No, not at all. Before, Taowu was the top dog...Lukou and Hengxi couldn't even compare with us. But now look, Taowu is gone, and we can't even be considered the third anymore.

From the interview findings, we can abstract a comparative mechanism: after being merged, Taowu lost its independent administrative status, leading to a weaker collective identity among its residents. As a result, they perceive their own development as lagging behind that of neighboring Lukou, giving rise to an upward social comparison. This perceived disparity, combined with Lukou's rapid growth, makes Taowu residents feel relatively dissatisfied with the TMP. Since Hengxi Subdistrict's development was considered inferior to that of Lukou, Taowu residents, now part of Hengxi, express weaker collective identification with their new administrative unit. This combination of low identity recognition and unfavorable social comparison ultimately contributes to Taowu's lower satisfaction. As previous studies have consistently demonstrated that upward social comparison tends to reduce residents' satisfaction and sense of recognition[45,46], particularly in the context of administrative adjustment and resettlement programs[46], our study further extends these findings to the scenario of township merger and regional restructuring.

Interestingly, residents of the former Hengxi town reported lower satisfaction with the TMP (Table 7), despite the fact that the two towns have now been merged into one subdistrict. In more detail, Taowu residents express higher policy satisfaction than those in Hengxi. A possible explanation lies in the relatively strong sense of identity among Hengxi residents, whose policy satisfaction is shaped primarily by temporal comparisons with their own past, particularly in the domains of environment and culture (Table 7). Although Hengxi outperforms Taowu in terms of employment incentives, its shortcomings in environmental and cultural outcomes contribute to its lower overall policy satisfaction. This pattern is consistent with our interview findings.

Q: "Are you satisfied with the development of Hengxi after the township merger?"

A: It's okay, I guess. The biggest thing is that Hengxi just isn't as lively as it used to be. We used to have opera troupes performing, and the March 17th fair would be packed with people...now that's all gone. The old street was such a wonderful place, but it's all run-down now.

In summary, residents in areas with weaker collective identity tend to compare themselves both with their own past and with more developed neighboring regions. This is a pattern often associated with lower levels of policy satisfaction. Meanwhile, residents with stronger collective identity would like to compare themselves with their own past

**Action Place:** "Discussion" Section

5. **The relatively limited sample size (N = 243) becomes even more problematic when the data are subdivided into subgroups based on age and region. Small subsamples, particularly in the elderly group, produce very large regression coefficients with wide standard errors, indicating potential instability of the estimates. Authors should address the issue of statistical power, provide robustness tests where possible, and provide explicit warnings about the limitations of interpreting the results of subgroup analyses.**

**Answer :** We greatly appreciate your valuable comments which are very insightful. To address the issue caused by small sample size, we first attempted the robustness checks you suggested. Specifically, we re-estimated the original subgroup regressions (categorized by age ~~and region~~) using the Bootstrap and Jackknife methods. The results confirmed that in small-sample subgroups such as the elderly group, the coefficients of the core independent variables became insignificant rendering the statistical inferences unreliable.

Considering that this subsection on subgroup analysis was originally intended as an extension of the baseline regression rather than as essential evidence supporting our core conclusions, and given that the current sample data cannot support stable estimations for such finely partitioned subgroups, we have decided to delete this subsection from the revised manuscript. We believe this removal helps avoid misleading interpretations and maintains the rigor of our study.

6. **Presenting logit regression results solely as logit coefficients limits readability and policy relevance, especially for readers across disciplines. The addition of marginal effects or predictive probabilities is highly recommended to clarify the substantive meaning and magnitude of policy impact.**

**Answer:** Thank you for your valuable suggestion. Since the logit model is nonlinear, its marginal effects depend on the values of the independent variables. To enhance interpretability, we have now included the marginal effects evaluated at the sample means in the regression results. These effects represent the change in the probability of reporting positive policy satisfaction associated with a one-unit change in an independent variable, holding all other variables constant.

**Action:** Column (5) of Table 6 presents the marginal effects of each variable at the sample mean. Specifically, when all other variables remain constant, a one-unit increase in the social security effect raises residents' satisfaction with the TMP policy by 70.5%; a one-unit increase in the cultural protection effect increases satisfaction by 69.4%; and a one-unit increase in the employment incentive effect boosts satisfaction by 34%.

**Table 6**

|  | **(1)** | **(2)** | **(3)** | **(4)** | **(5)** |
| --- | --- | --- | --- | --- | --- |
|  | Policy Satisfaction | Policy Satisfaction | Policy Satisfaction | Policy Satisfaction | Margin effects |
| Policy Satisfaction |  |  |  |  |  |
| Population Development Effect | 1.4119^**^ | 1.0650 | 1.0650 | 1.0650 | 0.259 |
|  | (0.7184) | (0.8544) | (1.0078) | (1.2489) | (0.211) |
| Infrastructure Effect | 0.4057 | 0.3068 | 0.3068 | 0.3068 | 0.075 |
|  | (2.0788) | (2.3137) | (1.0795) | (1.3809) | (0.562) |
| Environmental Improvement  Effect | 0.2285 | 0.6916 | 0.6916 | 0.6916 | 0.168 |
|  | (3.5689) | (3.2677) | (1.2713) | (1.4542) | (0.797) |
| Income Growth Effect | -0.6951 | -0.9425 | -0.9425 | -0.9425 | -0.229 |
|  | (1.2467) | (1.2393) | (0.7629) | (0.9470) | (0.304) |
| Employment Incentive Effect | 1.4578^***^ | 1.3958^**^ | 1.3958^*^ | 1.3958^***^ | 0.340^***^ |
|  | (0.5100) | (0.5538) | (0.7662) | (0.4712) | (0.131) |
| Social Security Effect | 2.2970^***^ | 2.8941^***^ | 2.8941^**^ | 2.8941^***^ | 0.705^***^ |
|  | (0.3480) | (0.0870) | (1.2555) | (1.0764) | (0.013) |
| Cultural Development Effect | 3.1524^**^ | 2.8488^**^ | 2.8488^***^ | 2.8488^***^ | 0.694^**^ |
|  | (1.2752) | (1.1858) | (0.8574) | (0.6170) | (0.281) |
| Gender |  | 0.1714 | 0.1714 | 0.1714 | 0.042 |
|  |  | (0.4103) | (0.3454) | (0.3117) | (0.099) |
| Age_18 |  | 0.6904 | 0.6904 | 0.6904 | 0.168 |
|  |  | (0.8645) | (0.7404) | (0.8243) | (0.209) |
| Age_31 |  | 0.5499^*^ | 0.5499 | 0.5499 | 0.134^*^ |
|  |  | (0.3179) | (0.6105) | (0.6745) | (0.076) |
| Age_46 |  | 0.6235^**^ | 0.6235 | 0.6235 | 0.152^**^ |
|  |  | (0.2798) | (0.5281) | (0.5376) | (0.070) |
| Occupation_Agriculture |  | -0.3812 | -0.3812 | -0.3812 | -0.093 |
|  |  | (0.5998) | (0.6477) | (0.9345) | (0.147) |
| Occupation_Firm |  | 0.1475^***^ | 0.1475 | 0.1475 | 0.036^***^ |
|  |  | (0.0154) | (0.6106) | (0.7250) | (0.003) |
| Occupation_Government |  | 0.5716 | 0.5716 | 0.5716 | 0.139 |
|  |  | (0.3813) | (0.8432) | (0.8372) | (0.091) |
| Occupation_Teaching |  | -1.2339 | -1.2339 | -1.2339 | -0.300 |
|  |  | (0.8246) | (0.9029) | (1.3728) | (0.197) |
| Occupation_Free |  | -0.2226 | -0.2226 | -0.2226 | -0.054 |
|  |  | (0.4515) | (0.5881) | (0.7847) | (0.109) |
| ln_time |  | 0.1699^*^ | 0.1699 | 0.1699 | 0.041^**^ |
|  |  | (0.0883) | (0.2229) | (0.3068) | (0.021) |
| _cons | -5.0998^***^ | -6.5936^***^ | -6.5936^***^ | -6.5936^***^ |  |
|  | (0.0883) | (1.2857) | (1.6776) | (1.4926) |  |
| N | 243 | 243 | 243 | 243 |  |
| pseudo R^2^ | 0.285 | 0.306 | 0.306 | 0.306 |  |

Note:* p < 0.1, ** p < 0.05, *** p < 0.01

**Action place:** the "Results" section

7. **Given the central role of Fairlie decomposition in drawing conclusions, authors should acknowledge the method's sensitivity to model specification, choice of comparator, and variable ordering. Including sensitivity analysis or alternative specifications would significantly enhance the credibility of the results.**

**Answer：**Thank you for your suggestion. We fully acknowledge the Fairlie decomposition method's potential sensitivity to model specification, choice of comparator, and variable ordering, and have supplemented three sets of sensitivity analyses to enhance result credibility.

(1) The results of the Fairlie decomposition are sensitive to the choice of reference group. When comparing differences between two regions, the decomposition results are inconsistent depending on which region is selected as the reference. Therefore, following Best et al. (2025), to avoid potential bias arising from the choice of reference group, we use coefficients estimated from the pooled sample of residents from both regions as the baseline for comparison.
(2) As mentioned in the "Research Design" section, within the explained component, the contribution of any specific variable, such as $X_{1}$ , to the overall difference can be expressed as:

$\sum_{i=1}^{n^{l}} \frac{F\left( {X_{1}}^{l}{\beta_{1}}^{t}+{{other}_{i}}^{l}{\beta_{other}}^{t} \right)}{n^{l}}$-$\sum_{i=1}^{n^{t}} \frac{F\left( {X_{1}}^{t}{\beta_{1}}^{t}+{{other}_{i}}^{t}{\beta_{other}}^{t} \right)}{n^{t}}$

The mean difference in policy satisfaction regarding the township-town merger between Lukou ($l$) and Taowu ($t$) is decomposed into two components, where $n^{l} and n^{t}$ denote the sample sizes of residents surveyed in the two regions, respectively, and $\text{β}^{\text{t}}$ represents the coefficients estimated from the pooled sample. Therefore, for any given variable, the order in which variables are introduced does not affect its explained contribution.

(3) Model specification: We re-estimated Fairlie decomposition using probit model. The results are shown in the Supporting Information (S4-6) with no substantial changes in significance or coefficient direction, and the fluctuation in contribution ratio did not exceed 5%.

**Action**：To mitigate the potential influence of benchmark selection, this study follows Fairlie's approach by using the estimated coefficients derived from the pooled sample of residents from both Subdistricts as $\beta^{t}$ in the first term on the right-hand side. Moreover, within the explained component, the contribution of any specific variable, such as $X_{1}$,to the overall difference can be expressed as:

$\sum_{i=1}^{n^{l}} \frac{F\left( {X_{1}}^{l}{\beta_{1}}^{t}+{{other}_{i}}^{l}{\beta_{other}}^{t} \right)}{n^{l}}$-$\sum_{i=1}^{n^{t}} \frac{F\left( {X_{1}}^{t}{\beta_{1}}^{t}+{{other}_{i}}^{t}{\beta_{other}}^{t} \right)}{n^{t}}$ (3)

Furthermore, this study re-examines the Fairlie decomposition using a probit model, with results presented in Supporting Information (S4-6). The results show that no substantial changes in significance or coefficient direction, and the fluctuation in contribution ratio did not exceed 5%.

**Action place：**Questionnaire design and data collection和 results部分

8. **There are several minor issues that also need attention, including the tendency to generalize findings outside the institutional and political context of China, the lack of transparency regarding the procedures for selecting respondents and analyzing qualitative data, inconsistencies in economic terminology, and descriptive redundancy in the background section of the study area.**

**Answer:** Thank you for your valuable suggestion.

(1) Tendency to generalize findings outside the **institutional and political context of China:** we have carefully refined the discussion of our conclusions to strike a balance between acknowledging the specific institutional context of China and articulating the broader implications of our research. We have not only clarified that our findings are situated within China's unique political and administrative system, but also argued that our findings could offer meaningful practical implications beyond China in administrative boundary adjustment policies if employed with caution.

As a specific form of administrative boundary reform, Township-town Merger Policy (similar policies with different names) has been implemented in many countries (Kössler K, Fessha Y T, 2025). Our study, therefore, contributes to the theoretical understanding of how such territorial reforms influence citizen satisfaction. Mechanisms like collective identity preservation may also be relevant in other contexts where administrative boundaries are redrawn, even if the institutional settings vary.

**Action: (1)** On a theoretical level, considering that the theoretical summary presented in this paper is based on China's political system, it cannot be used as a theoretical framework to guide changes in national boundaries in countries with different political structures. However, for other regions of China and countries with similar national conditions, this study makes theoretical contributions in three aspects.

(1) In practice, some successful experiences and concrete measures adopted by the two Subdistricts can offer useful insights for countries with different political systems during their boundary changes. For example, after the TMP, Lukou did not focus solely on economic efficiency, but consistently prioritized cultural preservation and the cultivation of community identity. As a result, Lukou achieved both economic growth and high resident satisfaction. This outcome echoes the observation by Kössler and Fessha (2025), who highlighted the ongoing challenge in African and European countries of balancing improved resource allocation through mergers or splits with the need to preserve local identity during boundary changes.

**Action Place：**(1) in "Theoretical implications"; (2) in "Practical implications"

**Answer:** (2) T**he lack of transparency regarding the procedures for selecting respondents and analyzing qualitative data:** To address this problem, we supplement the section "Questionnaire Design and Data Collection" by clarifying the sampling methods and qualitative data analysis process. Moreover, the interview transcripts are specifically provided in the interpretation of the results.

**Action:** Within each site, respondents were randomly selected to ensure representativeness. Respondents were eligible if they had resided in the subdistrict for at least 5 years to ensure familiarity with the TMP's long-term effects and were 18 years or older. 20 face-to-face interviews were analyzed using thematic analysis. Interview recordings were transcribed verbatim into text by apps. Researchers independently coded the text to identify key themes concerning identity recognition, expectancy formation, and social comparison.

**Acion Place:** Add a paragraph in "Questionnaire Design and Data Collection" section

**Answer:** (3) I**nconsistencies in economic terminology:** To address this issue, we read through the entire text and correct inconsistent economic terminology. For example, we unify "economic development effect" as "economic growth effect" to be consistent with Variable Definition Table 2, and unify "tangible economic benefits" as "direct economic benefits" to align with the expression in Literature Review.

**Answer:** (4) D**escriptive redundancy in the background section of the study area:** We have streamlined Section "Overview of the study area" by merging repetitive information, such as redundant emphasis on "geographical and cultural similarity" and deleting irrelevant details like the introduction of Nanjing city and non-essential airport location information etc.

***Reviewer #2 :***

1. **Some conclusions, especially those related to "collective identity" and "social comparison," are not directly measured in the statistical models and should be clearly presented as interpretive or qualitative explanations rather than as direct statistical results.**

**Answer :** Thank you for your suggestions. **First,** in stead of directly measuring psychological mechanisms such as identity, expectation formation, and social comparison, we analyzed these mechanisms based on qualitative data from post-survey interviews. As described in Section "Questionnaire design and data collection" and "Analysis of the determinants of policy satisfaction", we conducted random offline interviews with respondents after the questionnaire survey. Drawing on these interviews, we explored the reasons underlying regional differences in residents' policy satisfaction.

**Second,** the existing literature supports the validity of deriving psychological mechanisms from interview data. As emphasized by Mettler & Soss (2004), qualitative evidence, such as citizen interviews, is a critical complement to quantitative analysis in public policy research, enabling the unpacking of the "how" and "why" behind observed patterns. Consistent with this, Rahmqvist & Bara (2010) and Fiorio & Florio (2011) both integrated interview insights to explain the psychological drivers of policy satisfaction (e.g., identity and social comparison), demonstrating that interviews can effectively capture subjective mechanisms that are not directly measurable via questionnaires.

**Moreover,** social identity theory (Tajfel & Turner, 1979) and social comparison theory (Wills, 1981), which are cited in our literature review, provide theoretical frameworks that align with our interview-derived interpretations, further validating their plausibility.

**Third,** in the original manuscript, both empirical findings and theoretical interpretations were placed in the "Results" section, which may have compromised the readability of the mechanism analysis. Therefore, we added a "Discussion" section to the revised manuscript to explicitly link the mechanisms to interview evidence and theoretical interpretation, while leaving the "Results" section solely for presenting statistical findings.

**Action: Psychological mechanisms**

In the comparison between Taowu and Lukou, there is a substantial gap in policy satisfaction (Table 6). This indicates that Taowu underperforms compared to Lukou in overall economic aspects, especially in population development, social security, and employment incentives. On one hand, these findings are consistent with Lai[12], who discovered that township mergers led to notable population agglomeration, which ultimately enhances residents' policy satisfaction. On the other hand, the interviews also reveal that in Lukou, not only is the population more concentrated, but the area has also attracted numerous migrants who are satisfied with the TMP. For instance, one interviewee stated that her son chose to settle and work in Lukou because of the city's well-organized job fairs and effective job-seeking WeChat public accounts, which means good job opportunities. Meanwhile, interviews with the residents of Taowu show that they are dissatisfied with the significant difference in the development process between the two places.

Q: "Are you satisfied with the development of Taowu after the township merger?"

A: No, not at all. Before, Taowu was the top dog...Lukou and Hengxi couldn't even compare with us. But now look, Taowu is gone, and we can't even be considered the third anymore.

From the interview findings, we can abstract a comparative mechanism: after being merged, Taowu lost its independent administrative status, leading to a weaker collective identity among its residents. As a result, they perceive their own development as lagging behind that of neighboring Lukou, giving rise to an upward social comparison. This perceived disparity, combined with Lukou's rapid growth, makes Taowu residents feel relatively dissatisfied with the TMP. Since Hengxi Subdistrict's development was considered inferior to that of Lukou, Taowu residents, now part of Hengxi, express weaker collective identification with their new administrative unit. This combination of low identity recognition and unfavorable social comparison ultimately contributes to Taowu's lower satisfaction. As previous studies have consistently demonstrated that upward social comparison tends to reduce residents' satisfaction and sense of recognition[45,46], particularly in the context of administrative adjustment and resettlement programs[46], our study further extends these findings to the scenario of township merger and regional restructuring.

Interestingly, residents of the former Hengxi town reported lower satisfaction with the TMP (Table 7), despite the fact that the two towns have now been merged into one subdistrict. In more detail, Taowu residents express higher policy satisfaction than those in Hengxi. A possible explanation lies in the relatively strong sense of identity among Hengxi residents, whose policy satisfaction is shaped primarily by temporal comparisons with their own past, particularly in the domains of environment and culture (Table 7). Although Hengxi outperforms Taowu in terms of employment incentives, its shortcomings in environmental and cultural outcomes contribute to its lower overall policy satisfaction. This pattern is consistent with our interview findings.

Q: "Are you satisfied with the development of Hengxi after the township merger?"

A: It's okay, I guess. The biggest thing is that Hengxi just isn't as lively as it used to be. We used to have opera troupes performing, and the March 17th fair would be packed with people...now that's all gone. The old street was such a wonderful place, but it's all run-down now.

In summary, residents in areas with weaker collective identity tend to compare themselves both with their own past and with more developed neighboring regions. This is a pattern often associated with lower levels of policy satisfaction. Meanwhile, residents with stronger collective identity would like to compare themselves with their own past

**Action Place:** "Discussion" section

2. **The conclusions would also be stronger if key claims were more clearly linked to specific tables, such as the coefficients in Table 3 and the decomposition results in Table 7.**

**Answer:** Thank you for your suggestion. We fully agree with your comment. Following your advice, we have made some adjustments. **First**, when discussing the determinants of policy satisfaction, we tie the coefficient values in Table 3 to indicate the intensity of the effects. Table 7 (Table 6 in the revised manuscript) mainly shows the contribution ratio of each variable to the satisfaction gap between Lukou and Hengxi subdistricts. Therefore, when discussing the factors influencing regional disparities, we bind Table 7 (Table 6 in the revised manuscript), Contribution Proportion, to demonstrate the importance ranking of various factors.

**Second**, all references to Table 3 and Table 7 (Table 6 in the revised manuscript) have been marked as "Table 3" "Table 7" (Table 6 in the revised manuscript). We make sure that when readers see the conclusions, they can quickly locate the corresponding data tables.

**Third**, we also modified the table results in other sections.

3. There are also minor statistical points that need clarification. The manuscript reports clustered standard errors at the Subdistrict level, but the analysis compares only two Subdistricts, which may affect the reliability of significance levels.

**Answer:** Thank you for your valuable suggestion regarding the clustered standard errors. To address this concern and enhance the reliability of our significance levels, we conducted additional analyses. **First**, we re-estimated our models using ordinary standard errors as a robustness check. While the significance levels changed slightly, the coefficients for the employment incentive effect, social security effect, and cultural development effect remained positive and significant at the 10% level, consistent with the results obtained using clustered standard errors at the subdistrict level.

**Second**, to address the potential issue of underestimated standard errors due to clustering at the subdistrict level with only two clusters, we employed two-way clustering at the subdistrict and time levels. This approach accounts for potential correlations among individuals within the same subdistrict and at the same time point. Importantly, two-way clustering increased the number of clusters to seven, thereby improving the reliability of the clustered standard errors. The results, presented in Table 1, show that the coefficients for the employment incentive effect, social security effect, and cultural development effect remain positive and significant at the 10% level, consistent with our original findings.

**Table 1**

|  | **(1)** | **(2)** | **(3)** | **(4)** | **(5)** |
| --- | --- | --- | --- | --- | --- |
|  | Policy Satisfaction | Policy  Satisfaction | Policy  Satisfaction | Policy  satisfaction | Margin effects |
| Population Development Effect | 1.4119^**^ | 1.0650 | 1.0650 | 1.0650 | 0.259 |
|  | (0.7184) | (0.8544) | (1.0078) | (1.2489) | (0.211) |
| Infrastructure Effect | 0.4057 | 0.3068 | 0.3068 | 0.3068 | 0.075 |
|  | (2.0788) | (2.3137) | (1.0795) | (1.3809) | (0.562) |
| Environmental Development  Effect | 0.2285 | 0.6916 | 0.6916 | 0.6916 | 0.168 |
|  | (3.5689) | (3.2677) | (1.2713) | (1.4542) | (0.797) |
| Income Growth  Effect | -0.6951 | -0.9425 | -0.9425 | -0.9425 | -0.229 |
|  | (1.2467) | (1.2393) | (0.7629) | (0.9470) | (0.304) |
| Employment Incentive Effect | 1.4578^***^ | 1.3958^**^ | 1.3958^*^ | 1.3958^***^ | 0.340^***^ |
|  | (0.5100) | (0.5538) | (0.7662) | (0.4712) | (0.131) |
| Social Security Effect | 2.2970^***^ | 2.8941^***^ | 2.8941^**^ | 2.8941^***^ | 0.705^***^ |
|  | (0.3480) | (0.0870) | (1.2555) | (1.0764) | (0.013) |
| Cultural Development Effect | 3.1524^**^ | 2.8488^**^ | 2.8488^***^ | 2.8488^***^ | 0.694^**^ |
|  | (1.2752) | (1.1858) | (0.8574) | (0.6170) | (0.281) |
| Gender |  | 0.1714 | 0.1714 | 0.1714 | 0.042 |
|  |  | (0.4103) | (0.3454) | (0.3117) | (0.099) |
| Age_18 |  | 0.6904 | 0.6904 | 0.6904 | 0.168 |
|  |  | (0.8645) | (0.7404) | (0.8243) | (0.209) |
| Age_31 |  | 0.5499^*^ | 0.5499 | 0.5499 | 0.134^*^ |
|  |  | (0.3179) | (0.6105) | (0.6745) | (0.076) |
| Age_46 |  | 0.6235^**^ | 0.6235 | 0.6235 | 0.152^**^ |
|  |  | (0.2798) | (0.5281) | (0.5376) | (0.070) |
| Occupation_Agriculture |  | -0.3812 | -0.3812 | -0.3812 | -0.093 |
|  |  | (0.5998) | (0.6477) | (0.9345) | (0.147) |
| Occupation_Firm |  | 0.1475^***^ | 0.1475 | 0.1475 | 0.036^***^ |
|  |  | (0.0154) | (0.6106) | (0.7250) | (0.003) |
| Occupation_Government |  | 0.5716 | 0.5716 | 0.5716 | 0.139 |
|  |  | (0.3813) | (0.8432) | (0.8372) | (0.091) |
| Occupation_Teaching |  | -1.2339 | -1.2339 | -1.2339 | -0.300 |
|  |  | (0.8246) | (0.9029) | (1.3728) | (0.197) |
| Occupation_Free |  | -0.2226 | -0.2226 | -0.2226 | -0.054 |
|  |  | (0.4515) | (0.5881) | (0.7847) | (0.109) |
| ln_time |  | 0.1699^*^ | 0.1699 | 0.1699 | 0.041^**^ |
|  |  | (0.0883) | (0.2229) | (0.3068) | (0.021) |
| _cons | -5.0998^***^ | -6.5936^***^ | -6.5936^***^ | -6.5936^***^ |  |
|  | (0.0883) | (1.2857) | (1.6776) | (1.4926) |  |
| N | 243 | 243 | 243 | 243 |  |
| pseudo R^2^ | 0.285 | 0.306 | 0.306 | 0.306 |  |

**注：*** p < 0.1, ** p < 0.05, *** p < 0.01.Column 1 and Column 2 use cluster-level regional mislabeling, Column 3 uses standard error, and Column 4 uses a dual standard error for both region and time.

**Action:** Column (3) reports the ordinary standard errors, while column (4) reports the two-way clustered standard errors at the regional and temporal levels. The results still indicate that the employment incentive effect, social security effect, and cultural development effect all exhibit a significantly positive correlation with policy satisfaction.

**Action place:** "Results" section

4. **In addition, the type of R² reported for the logit models should be clarified, and some subgroup results appear unstable due to small sample sizes.**

**Answer:** Thank you for your suggestion. Regarding the R² in Logit models, we clarify that, unlike linear regression where R² directly measures variance explained, Logit models use a nonlinear goodness-of-fit metric. Our reported values are McFadden's pseudo R², the standard, widely accepted measure for such nonlinear discrete-choice models.

‌Pseudo R² =$1-\frac{\ln l(Mfull)}{\ln l(Mintercept)}$, where $\ln l(Mfull)$ denotes the log-likelihood value of the full model incorporating all independent variables, and $\ln l(Mintercept)$ represents the log-likelihood value of the intercept-only model (containing only the constant term). The core logic of McFadden's pseudo R² lies in comparing the log-likelihood values of the full model (Mfull) and the intercept-only model (Mintercept). Specifically, if the full model significantly improves the log-likelihood value (i.e., fits the data better), the pseudo R² value will be higher, indicating a better model fit.

To avoid any misunderstanding, we have revised the relevant Tables by replacing the original label of R² with pseudo R².

To address the issue caused by small sample size, we re-estimated the original subgroup regressions (categorized by age) using the Bootstrap and Jackknife methods. The results confirmed that in small-sample subgroups such as the elderly group, the coefficients of the core independent variables became insignificant, rendering the statistical inferences unreliable.

Considering that this subsection on subgroup analysis was originally intended as an extension of the baseline regression rather than as essential evidence supporting our core conclusions, and given that the current sample data cannot support stable estimations for such finely partitioned subgroups, we have decided to delete this subsection from the revised manuscript. We believe this removal helps avoid misleading interpretations and maintains the rigor of our study.

**References:**

1．Gu, H.; Liu, Y.; Qian, F.; Wang, Q.; Dong, X. (2021). An empirical analysis of the factors affecting farmer satisfaction under the China Link Policy. *Sage Open*. 11, 21582440211023204.
2．Liu, I. C. (2014). Evaluation of Taiwan's National Health Insurance policy: An importance–satisfaction analysis. *The International Journal of Health Planning and Management*, 29(2), e145-e158.
3．Wang, W., Zhou, L., Yang, G., Sun, Y., & Chen, Y. (2019). Prohibited grazing policy satisfaction and life satisfaction in rural Northwest China. International Journal of Environmental Research and Public Health, 16(22), 4374.
4．Pelletier, L. G., Legault, L. R., & Tuson, K. M. (1996). The environmental satisfaction scale: A measure of satisfaction with local environmental conditions and government environmental policies. Environment and Behavior, 28(1), 5–26.
5．Helliwell, J. F., Layard, R., Sachs, J. D., De Neve, J. E., Aknin, L. B., & Wang, S. (2026). Why single-item measures of wellbeing are best. *Nature Human Behaviour*, 1-2. <https://doi.org/10.1038/s41562-026-02401-y)>
6．Van Ryzin, G. G. (2004). Expectations, performance, and citizen satisfaction with urban services. Journal of Policy Analysis and Management, 23(3), 433-448.
7．Rahmqvist, M., & Bara, A. C. (2010). Patient characteristics and quality dimensions related to patient satisfaction. *International Journal for Quality in Health Care*, 22(2), 86-92.
8．Fiorio, C. V., & Florio, M. (2011). Would you say that the price you pay for electricity is fair? Consumers' satisfaction and utility reforms in the EU15. Energy Economics, 33, 178-187.
9．Jilke, S., & Van De Walle, S. (2013). Two track public services? Citizens' voice behaviour towards liberalized services in the EU15. Public Management Review, 15(4), 465-476.
10．Andersson, L. M., & Bateman, T. S. (1997). Cynicism in the workplace: Some causes and effects. *Journal of Organizational Behavior: The International Journal of Industrial, Occupational and Organizational Psychology and Behavior*, 18(5), 449-469.
11．Aulakh, P. S., & Gencturk, E. F. (2000). International principal-agent relationships-control, governance and performance. *Industrial Marketing Management*, 29, 521-538.
12．Gong, Y., Li, J., Xie, J., Zhang, L., & Lou, Q. (2022). Will "green" parents have "green" children? The relationship between parents' and early adolescents' green consumption values. *Journal of Business Ethics*, 179(2), 369-385.
13．Podsakoff, P. M., MacKenzie, S. B., Lee, J. Y., & Podsakoff, N. P. (2003). Common method biases in behavioral research: a critical review of the literature and recommended remedies. *Journal of Applied Psychology*, 88(5), 879.
14．Mettler, S.; Soss, J. (2003). The consequences of public policy for democratic citizenship: bridging policy studies and mass politics. *Perspectives on Politics*. 2, 55.
15．Tajfel, H.; Turner, J. C. (1979). An integrative theory of intergroup conflict. In the social psychology of intergroup relations. W. G. Austin., S. Worchel., Eds.; *Brooks/Cole: Monterey, CA*. 33-37.
16．Wills, T. A. (1981). Downward comparison principles in social psychology. *Psychological Bulletin*. 90, 245.
17．Best, R., Chareunsy, A., & Nazifi, F. (2025). Persistent energy poverty for renters motivates policy reform. *Energy Economics*, 147, 108577.
18．Kössler, K., & Fessha, Y. T. (2025). The (re) making of local boundaries in African and European federal systems: Arguments and intergovernmental dynamics. *Publius: The Journal of Federalism*, pjaf014.
